# Head Rubbing and Licking Reinforce Social Bonds in a Group of Captive African Lions, *Panthera leo*

**DOI:** 10.1371/journal.pone.0073044

**Published:** 2013-09-04

**Authors:** Tomoyuki Matoba, Nobuyuki Kutsukake, Toshikazu Hasegawa

**Affiliations:** 1 Department of Cognitive and Behavioral Science, Graduate School of Arts and Sciences, The University of Tokyo, Tokyo, Japan; 2 Department of Evolutionary Studies of Biosystems, The Graduate University for Advanced Studies, Hayama, Japan; 3 Precursory Research for Embryonic Science and Technology (PRESTO), Japan Science and Technology Agency, Kawaguchi, Japan; Bangor University, United Kingdom

## Abstract

Many social animals have a species-specific repertoire of affiliative behaviours that characterise individualised relationships within a group. To date, however, quantitative studies on intragroup affiliative behaviours in social carnivores have been limited. Here, we investigated the social functions of the two most commonly observed affiliative behaviours in captive African lions (*Panthera leo*): head rubbing and licking. We conducted behavioural observations on a captive group of lions composed of 7 males and 14 females, and tested hypotheses regarding three social functions: tension reduction, social bonding, and social status expression. Disproportionately frequent male–male and female-to-male head rubbing was observed, while more than 95% of all licking interactions occurred in female–female dyads. In accordance with the social bond hypothesis, and in disagreement with the social status expression hypothesis, both head rubbing and licking interactions were reciprocal. After controlling for spatial association, the dyadic frequency of head rubbing was negatively correlated with age difference while licking was positively correlated with relatedness. Group reunion after daily separation did not affect the frequencies of the affiliative behaviours, which was in disagreement with the predictions from the tension reduction hypothesis. These results support the social bond hypothesis for the functions of head rubbing and licking. Different patterns of affiliative behaviour between the sexes may reflect differences in the relationship quality in each sex or the differential predisposition to licking due to its original function in offspring care.

## Introduction

Many social animals have a species-specific behavioural repertoire of affiliative interactions. These behaviours are usually non-randomly distributed within a group and are affected by factors such as individual traits (e.g. sex and body size), states (e.g. age, dominance rank and reproductive state) and behavioural context. Animals across a wide range of taxa use ritualised non-agonistic behaviours to manage intragroup social relationships to maintain social bonds with valuable partners [1]–[4], reduce tension [3], [5]–[8], and express social status (submission and dominance) within a group [9]–[11]. To date, quantitative studies of intragroup affiliative behaviours based on fine-scale behavioural observations of social carnivores have been limited to a few species, such as spotted hyenas [4], [12], [13], coatis [14], [15], and meerkats [16], [17].

Studies on social interactions in felids are even scarcer due to their solitary nature. Inconsistent reports exist regarding the function of allogrooming in domestic cats. Curtis et al. [18] found that male and female cats in captivity directed allogrooming toward familiar and related individuals, suggesting its social function to establish and maintain affiliative relationships. On the other hand, in a similar captive setting, van den Bos [19] observed that dominant cats (mostly males) groomed subordinates more frequently than they received grooming from subordinates, often with aggression and regardless of kinship, which suggests that allogrooming can be a mild form of aggression. In long-term coalitions of wild male cheetahs composed of brothers or unrelated individuals, allogrooming is distributed equally [20]. In captivity, many other felid species, despite having solitary lifestyles in the wild, express rubbing and licking behaviour toward their keepers [21], which indicates that rubbing and licking are common in the felid behavioural repertoire. Lions, which live in groups with a unique social structure and express a set of social interactions, have not been the subjects of such behavioural studies.

The lion social system exhibits considerable intraspecific variation across the species’ range [22], [23]. Lion sociality has been well documented by a long-term field research project in the Serengeti ecosystem (e.g. [24], [25]). A pride, the basic social unit of lions, is typically composed of 2–9 (maximum 21) related females, their offspring, and a coalition of 1–6 (maximum 9) males that are unrelated to the females [26], [27]. Unrelated males can form coalitions of two to three individuals, but larger coalitions are composed of close kin [26]. Lions form a fission–fusion society in which members travel in subgroups of variable composition [24], [28]. Multiple females in a pride give birth simultaneously and young cubs are nursed communally [29]. Dispersal is male-biased, with most females remaining in the natal pride while cohort males form a coalition and leave the natal pride to become nomadic. Nomadic male coalitions may fight with resident males to acquire a territory and reproductive opportunities. Takeover of a pride is usually followed by infanticide [30]. Male reproductive success is evenly distributed in small coalitions, but becomes skewed as coalitions become larger [26].

**Figure 1:**
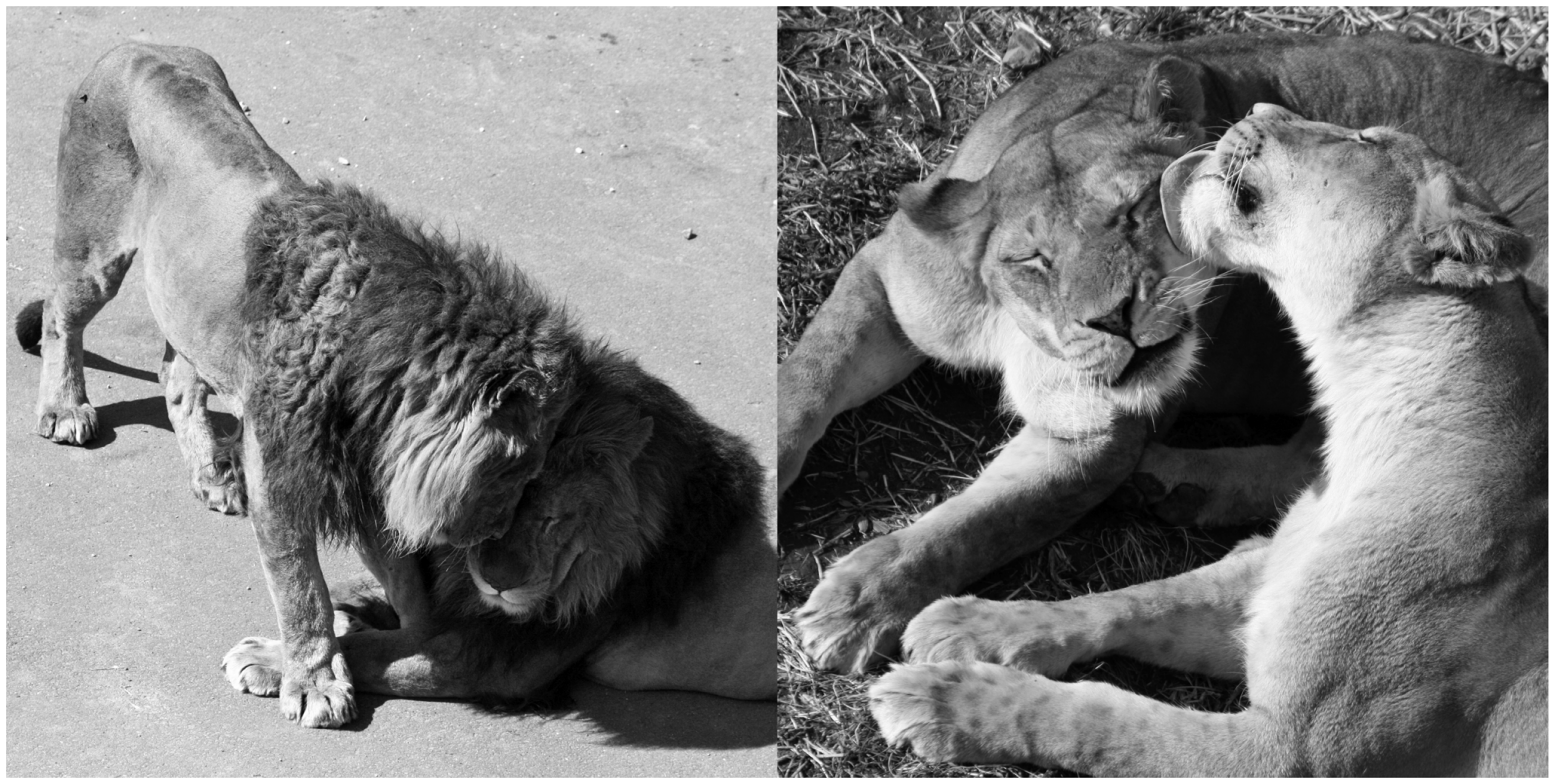
Pictures of lion affiliative interactions. A male rubs its head against the forehead of a resting male (left) and a female licks another female’s face (right).

Previous research on lion social behaviour has mainly focused on intergroup conflict (e.g. [31]), while intragroup social interactions in other contexts have attracted little attention. The two most conspicuous affiliative behaviours are head rubbing and licking ([24]; Figure. 1). Head rubbing is described as follows: “one (lion) bends its head toward the other’s head or neck, or, more probably, under its chin” [32]. Head rubbing can last for more than 1 min, but it can also occur in an abbreviated form with a slight bending of the head toward the other lion. In addition to providing tactile stimulation, head rubbing may also act as an olfactory form of communication (i.e., picking up and/or depositing scent). Odours play an important role in the social life of lions and felids in general [33]. When scent marking with urine, a lion either scratches the ground immediately after squatting down to urinate, or sprays urine over objects while in an upright position and with a vertically raised tail [24]. Prior to this scent marking, they often rub their head over the object, and in a recent study, volatile organic compounds from the faces of lions and other large felids were identified [34]. In addition, lions may acquire information on individual identity and reproductive condition by sniffing the hindquarters of the conspecifics they encounter [24]. Whether olfactory stimuli induce estrus synchrony in females is unknown. Licking part of another individual’s body, also referred to as grooming, is considered to have social as well as hygienic benefits [24], [32], in the same way as grooming in primates [35], [36] and other mammalian taxa [16], [37]. Other affiliative behaviours exhibited by lions include social play in which juveniles and occasionally adults also engage. Less conspicuously, some vocalisation and physical contact while resting may also convey amicable intention [24], although detailed investigations are lacking.

In this study, we quantitatively described the distributions of these affiliative behaviours in a captive group of lions and tested three hypotheses regarding their social functions. These hypotheses are not mutually exclusive, and thus the behaviours could have multiple functions.

### Hypothesis 1: Tension Reduction

Social stress caused by instability in relationships may eventually disrupt cooperation among group members. To cope with relationship uncertainty, mammals living in fission–fusion societies exchange greeting behaviours that quickly update relationships (chimpanzees: [38]; bonobos: [5]; spider monkeys: [6]; spotted hyenas: [4], [12]). Although it may be risky for an individual to approach and physically contact a conspecific with which it has an uncertain relationship, according to Schaller [24], head rubbing in lions often occurs after fights and other stressful events, and when dissociated pride members reunite. We hypothesised that head rubbing functions to moderate tension by reducing the aggressive intent of an interaction partner. A considerable amount of evidence indicates that grooming in primates, which serves a hygienic function equivalent to licking in lions, promotes tension reduction [39], [40]. The tension reduction hypothesis leads to the following predictions:

Prediction 1.1: When individuals are separated and their interactions are limited, the stability of their relationship declines. Therefore, head rubbing and licking should be frequent during encounters after separation (i.e. when animals exit the indoor enclosure and reunite in the morning).

Prediction 1.2: Two individuals with low baseline social interaction rate may experience stress when they are in proximity to each other. Communal cub rearing by lionesses [29] is likely to result in frequent affiliation among same- and adjacent-aged cubs, which may develop into lasting bonds. In contrast, less social interaction is expected in dyads with larger age differences. Hence, after controlling for spatial association, the frequencies of head rubbing and licking should correlate positively with age difference.

### Hypothesis 2: Social Bond

Social bonds between two conspecific animals can be defined by the occurrence of disproportionally frequent affiliative behaviours among them compared to other dyads within the group [41], [42]. In addition, affiliative behaviours can be viewed as investments in the development of social bonds [43]. When ecological or social conditions enhance or diminish a particular class of an individual’s value as a social partner, conspecific group members alter the amount of affiliative behaviour expressed toward that individual accordingly [17], [44]. Schaller [24] argued that head rubbing functions to unite the pride and strengthen social bonds. In the life history of lions, relationships between adult males and females last for the short period of male residency in a pride [45], while relationships between same-sex individuals last for a lifetime. Male coalitions last through the nomadic period and pride residency. The longer residency of larger coalitions [46] suggests male bonding may have a positive effect on their reproductive success. In philopatric females, relationships can continue throughout their lifetime unless pride fission occurs. Since larger prides can maintain higher-quality territories [25] and more effectively defend cubs against infanticidal males [47], females may also benefit from social bonds with other females. If head rubbing and licking have the function of maintaining these relationships, the following predictions can be made.

Prediction 2.1: Head rubbing and licking should be frequent among more strongly bonded individuals that (a) form same-sex dyads, (b) are highly related and (c) are similar in age.

Prediction 2.2: Head rubbing and licking should be exchanged reciprocally, since neither males nor females are reported to have linear dominance in rank. Reciprocal relationships should be unaffected even when immediate exchanges are excluded.

### Hypothesis 3: Expression of Social Status

The distributions of intragroup affiliative behaviours are often affected by the difference in dominance rank between individuals. In primates, for example, a general tendency for dominant individuals to receive more grooming than subordinates is observed [48]. There are two possible directions of social status expression: submission and dominance assertion. In carnivores, low-ranking domestic cats approach high-ranking conspecifics with their tails held vertically [11], and subordinate spotted hyenas show submission to dominants by exposing their genitals [12].

Female lions in a pride lack dominance rank system in relation to access to food [49], and although males in a large coalition have skewed reproductive success [26], they do not have dominance rank expressed by agonistic or submissive behavior [24]. Lions exhibit considerable sexual size dimorphism that gives males an advantage during physical contests with females. Schaller [24] associated this asymmetry in resource holding potential between the sexes with his observation that females direct more head rubbing to males than to other females. Submission and dominance predict opposite patterns in the direction of affiliative behaviours as follows.

Prediction 3.1 (a): If head rubbing and/or licking function as dominance assertion, male-to-female interactions should be more frequent than the inverse.

Prediction 3.1 (b): If head rubbing and/or licking function as submission, female-to-male interactions should be more frequent than the inverse.

Prediction 3.2: Since both dominance assertion and submission predict unidirectional interactions, head rubbing and licking should be non-reciprocal.

## Materials and Methods

### Ethics Statement

This study complies with Japanese regulations regarding the ethical treatment of research subjects. Research permission to conduct the study was granted by the Tokyo Zoological Park Society. This study was fully observational and our data collection did not affect the lions’ welfare.

### Subjects and Housing

**Figure 2:**
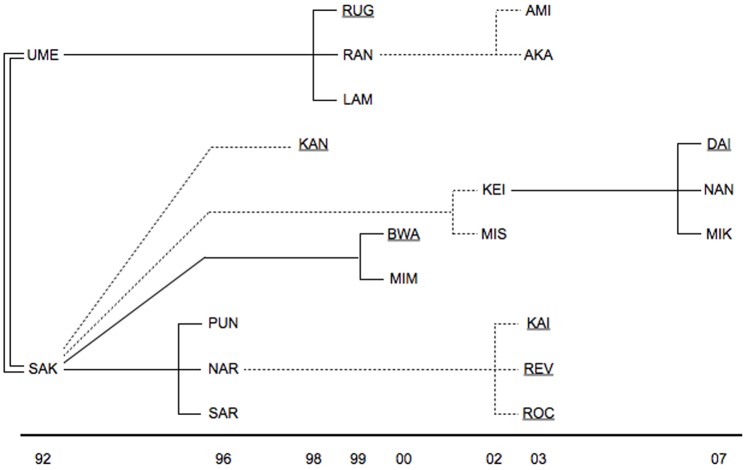
Kinship in the subject group of lions. Males are indicated by underlined IDs. Siblings from the same litter are connected by vertical lines. Bold, dashed and double lines represent three different sire males. Birth years are indicated at the bottom.

The subject of this study was a group of captive lions kept in the Tama Zoological Park, Tokyo, Japan. The group was composed of two founding adult female siblings that were introduced in 1994 and their offspring (Figure 2). During the observation period, the group included 21 individuals, including 6 adult males, 12 adult females, 1 subadult male, and 2 subadult females (adult: >4years, subadult: 2–4 years [24]; note that the subadults had reached body sizes comparable to that of adults). Individual identification was based on scars, coat colour, physique, and other natural features. Reproductive control was conducted on this group; all of the group males, including the subadults, were vasectomised and sires (not shown in Figure 2) were kept separately from the group and introduced to unrelated adult females for breeding every 2–3 years. The effect of vasectomy on social behaviour of male social carnivores is considered to be limited [50], [51]. Over the study period, no individuals showed stereotypical or abnormal behaviours. The average relatedness of the group, calculated from the zoo studbook, was 0.32 (range: 0.13–0.50). The average relatedness values among males and females were 0.31 (range: 0.13–0.50) and 0.33 (range: 0.13–0.50), respectively.

Lions were maintained in an outdoor enclosure (approximately 14 000 m^2^) between approximately 09∶50 and 16∶10 h, except during weekly park holidays. Depending on their physical conditions (e.g. injuries), some group members were not exhibited on a few days during the observation period. The enclosure contained natural vegetation, two artificial ponds, and three wooden feeding platforms and was partly paved with asphalt and concrete for the passage of a visitor bus. The bus usually ran a fixed route in the enclosure once every 15 min, or at shorter intervals when the park was busy, from 10∶00 until 16∶00 h with each round taking approximately 8 min. The lions were well habituated to the presence of the bus and showed no apparent behavioural aberrations when it was present. After the bus’ last round, the lions were housed in seven indoor enclosures (approximately 20 m^2^ each room) and were not exhibited for visitors, with 2–4 individuals of fixed membership being housed together every day. The animals were fed horsemeat and chicken heads three times a week in the indoor enclosure. Additionally, cow bones were provided on the feeding platforms in the outdoor enclosure and small amounts of meat were offered as bait attached to the bus. Water was available ad libitum in both the indoor and outdoor enclosures.

### Observation Methods

In total, 101 hours of behavioural observations were conducted over 27 days between 22 December 2008 and 24 March 2009. Using an all-occurrence method [52], we recorded the actor, the receiver, and the times of occurrence and termination of agonistic (biting, charging, lunging, chasing, growling and snarling; total n = 224, individual mean ± SE = 10.7±1.65, range = 2–28, dyad mean ± SE = 0.51±0.07, range = 0–19) and affiliative (head rubbing: total n = 514, individual mean ± SE = 24.5±2.3, range = 10–48, dyad mean ± SE = 1.22±0.13, range = 0–14; and licking: total n = 141, individual mean ± SE = 6.71±2.06, range = 0–35, dyad mean ± SE = 0.34±0.09, range = 0–19) behaviours. Additionally, through scan sampling conducted every 15 min, we recorded all of the dyads whose inter-individual distance was less than 2 m, allowing for the chain rule (i.e. if individual A is less than 2 m away from B and B is less than 2 m away from C, A and C are also considered to be in proximity). The total number of scans was 242 (daily mean ± SE = 10.5±0.74). Observations were conducted using 8 × 32 binoculars from viewpoints around and more than 5 m above the enclosure where zoo visitors also had access. Observation points were occasionally switched to maximise the number of visible subjects and to cover the majority of the enclosure.

### Data Analysis

We conducted the following analyses by both including and excluding subadult data; however, the results did not differ qualitatively. Therefore, we report the results of analyses that included subadults.

The association index (AI) was calculated for each dyad to control for the effect of proximity on the frequency of affiliative behaviours. Daily AI was calculated by dividing the number of scans when two individuals were observed in proximity by the total number of scans conducted on that observation day. By calculating daily means, we circumvented data biases that were likely to be caused by autocorrelation between temporally adjacent sampling points during which the locations of individuals may have been static. All daily AI values were then averaged to calculate an AI for that dyad during the observation period.

To determine whether the distributions of head rubbing and licking were biased to a particular sex class combination of actor and receiver, we conducted a chi-square test on the total number of affiliative behaviours between each sex class combination. We then calculated individual mean frequencies for affiliative behaviours directed to same-sex and opposite-sex individuals and conducted individual-level comparisons using Wilcoxon signed rank tests.

Agonistic behaviours were most frequently observed immediately after reunion (i.e., when lions were released into the outdoor enclosure) in the morning (Matoba et al., unpublished data). To determine if affiliative behaviours were also frequent during that period, we used a generalised linear mixed model (GLMM) with a Poisson error structure and a log-link function. The response variable was the frequency of affiliative behaviours (head rubbing and licking) and the explanatory variable was the time of occurrence (hours, continuous variable), considering the random effect of individuals.

To investigate reciprocity in affiliative behaviours and the correlations between the frequencies of affiliative behaviours and other dyadic variables (relatedness and age difference), we conducted a *Kr*-test [53], which is a variation of a matrix correlation analysis. This method avoids the problem of pseudo-replication by conducting row-wise permutations [53]. Therefore, it is possible to determine whether the frequency of one social interaction is correlated with another interaction within each actor individual, while at the same time controlling for individual variation in interaction rates. Since the *Kr*-test is a non-parametric test, the result is unlikely to be biased by outlier data. Half matrix data on relatedness, age difference and association were converted to full matrix data by adding a transposed half matrix for statistical analysis. To distinguish long-term reciprocity from immediate exchange, we also produced data sets excluding head rubbing and licking performed by the receiver to the actor of the previous interaction less than 10 min before, and we then re-ran the *Kr*-tests on those data sets. Although the time frame of immediate reciprocity perceived by the animals may have been longer than 10 min, we believe this is a conservative criterion because the excluded interactions occurred mostly within 2 min (3 of 4 head rubbing and 18 of 22 licking) of the previous interaction. All tests were two-tailed (significance level set to 0.05) based on a randomised distribution of 2000 permutations. To control for the effect of spatial proximity on affiliative behaviours and to eliminate the possibility that one correlation could arise as a by-product of another, we conducted partial *Kr*-tests [54] that separately controlled for three variables: relatedness, age difference and AI. MatrixTester version 2.2.0b^©^ by C. Hemelrijk was used to conduct the *Kr*-tests, and R version 2.11.1 [55] was used for all of the other analyses.

## Results

### Distribution of Head Rubbing and Licking among Sex Classes

**Figure 3:**
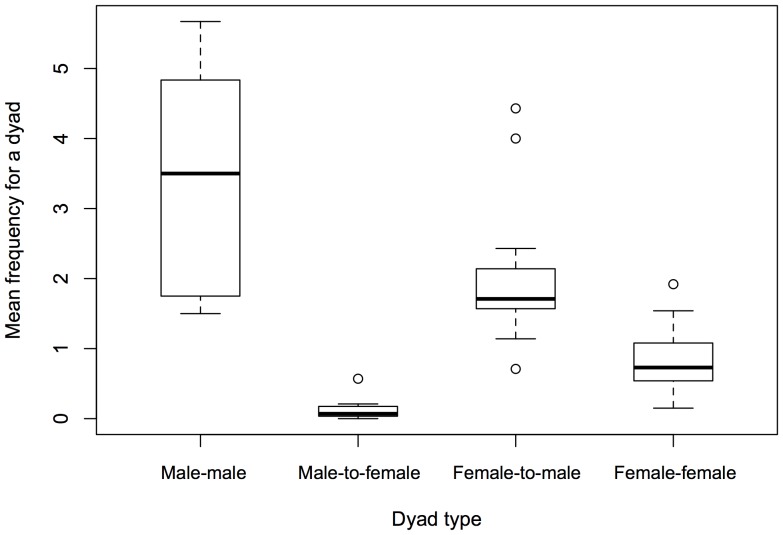
Boxplot of the frequency of head rubbing for each sex classs dyad. Bold lines indicate medians and circles denote outliers.

**Figure 4:**
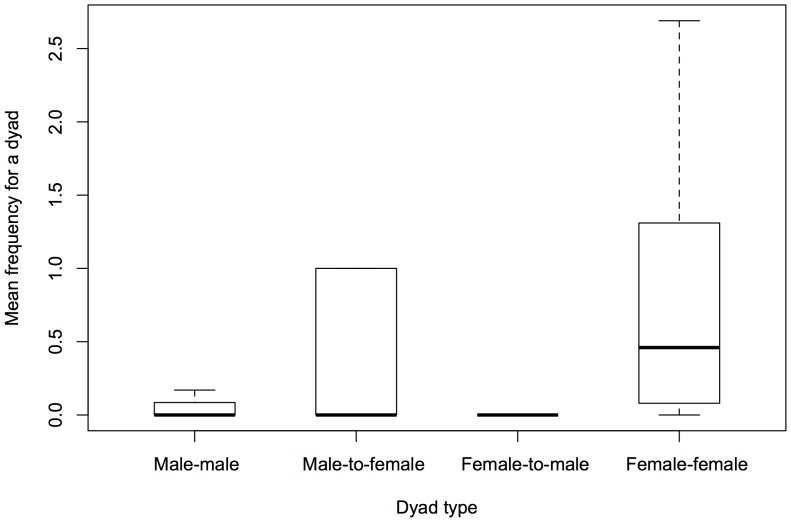
Boxplot of the frequency of licking for each sex classs dyad. Bold lines indicate medians.

The frequency distribution of head rubbing was affected by the sex classses of the actor and the receiver. The distribution of 514 observed head rubbing events differed significantly from the expected random distribution with actor–receiver sex (chi-square test using pooled data, χ^2^_1_ = 331, p<0.001). At the individual level, males directed significantly more head rubbing to other males than to females (mean ± SE = 3.40±0.51 vs. 0.15±0.05, Wilcoxon signed rank test, N = 7, T = 0, p = 0.018; Figure 3). Females directed significantly more head rubbing to males than to other females (mean ± SE = 2.04±0.19 vs. 0.86±0.10, Wilcoxon signed rank test, N = 14, T = 2, p = 0.002; Figure 3). Licking was mainly observed between females, of which 96.5% (136 of 141) occurred between lionesses (Figure 4). Therefore, we only analysed female–female licking.

### Effect of Spatial Proximity

A positive correlation was observed between the frequency of head rubbing and the AI of each dyad at the group level (*N = *21, *Kr = *692, *tau Kr* = 0.218, *p = *0.0005). Analysing each actor–receiver sex class separately, head rubbing frequency was positively correlated with the AI in all types of dyads (male–male: *N = *7, *Kr = *62, *tau Kr = *0.634, *p = *0.005; male-to-female: *N = *7 for row and 14 for column, *Kr = *50, *tau Kr = *0.505, *p = *0.002; female-to-male: *N = *14 for row and 7 for column, *Kr = *43, *tau Kr = *0.168, *p = *0.008; female–female: *N = *14, *Kr = *289, *tau Kr = *0.244, *p = *0.0005). Female licking frequency was positively correlated with the AI (*N = *14, *Kr = *176, *tau Kr = *0.288, *p = *0.002).

### Hypothesis Testing

Hypothesis 1: Tension reduction. Prediction 1.1 (not supported): Head rubbing did not occur more frequently after reunion, i.e. in the morning hours (GLMM, β = –0.013, SE = 0.031, Z = –0.42, p = 0.67). Licking more frequently occurred earlier in the day (GLMM, β = –0.18, SE = 0.063, Z = –2.87, p = 0.004). However, no licking was observed immediately after the lions were reunited in the outdoor enclosure, i.e. before 10∶00 h.

Prediction 1.2 (not supported): A negative correlation was observed between the frequency of head rubbing and the age difference of each dyad at the group level. The correlation remained significant after we controlled for the effect of relatedness and the AI. Analysing each actor–receiver sex classs separately, with the exception of male-to-female interactions, head rubbing frequency was negatively correlated with age difference and the results were not affected when we controlled for relatedness and the AI (Table 1). In addition, the results were not affected when we excluded the three subadult individuals. Female licking frequency was also negatively correlated with relatedness, but the correlation became non-significant after we controlled for the AI (Table 2).

**Table 1:** Results of *Kr*-tests: reciprocity of head rubbing and correlations with dyadic variables.

					-Relatedness						-Age difference										-Association index										
Overall (*N* = 21)		*Kr*	*tau Kr*	*p*	*tau Kr*				*p*				*tau Kr*					*p*					*tau Kr*				*p*				
Reciprocity		308	0.125	0.013*	0.116		0.016*				0.097						0.029* †				0.055				0.147						
Relatedness		274	0.096	0.0325*	–		–				0.043						0.192				0.057				0.127						
Age difference		–660	–0.222	0.001*	–0.205		0.0015*				–						–				–0.193				0.002*						
					**-Relatedness**						**-Age difference**										**-Association index**										
**Male–male (N = 7)**		***Kr***	***tau Kr***	***p***	***tau Kr***		***p***				***tau Kr***					***p***				***tau Kr***					***p***						
Reciprocity		18	0.200	0.096	0.196		0.109				0.123						0.216				0.019				0.463						
Relatedness		7	0.081	0.269	–		–				0.012						0.424				0.061				0.335						
Age difference		–53	–0.597	0.0065*	–0.593		0.003*				–						–				–0.518				0.006*						
					**-Relatedness**						**-Age difference**										**-Association index**										
**Male-to-female (N = 7 for row, 14 for column)**		***Kr***	***tau Kr***	***p***	***tau Kr***		***p***				***tau Kr***					***p***						***tau Kr***			***p***						
Reciprocity		36	0.391	0.0005*	0.375				0.003*				0.411						0.001*				0.360					0.002*			
Relatedness		25	0.285	0.026*	–				–				0.314						0.0135*				0.207					0.113			
Age difference		6	0.065	0.256	0.150				0.440				–						–				0.077					0.305			
					**-Relatedness**								**-Age difference**										**-Association index**								
**Female-to-male (N = 14 for row, 7 for column)**		***Kr***	***tau Kr***	***p***	***tau Kr***	***p***				***tau Kr***					***p***							***tau Kr***		***p***							
Reciprocity		36	0.391	0.0005*	0.375			0.003*				0.411						0.001*				0.360					0.002*				
Relatedness		27	0.121	0.075	–			–				0.077						0.159				0.088					0.136				
Age difference		–44	–0.184	0.008*	–0.159			0.023*				–						–				–0.186					0.012*				
					**-Relatedness**							**-Age difference**										**-Association index**									
**Female–female (N = 14)**	***Kr***		***tau Kr***	***p***	***tau Kr***				***p***					***tau Kr***				***p***			***tau Kr***					***p***					
Reciprocity		166	0.254	0.001*	0.227				0.001*				0.140						0.0295*				0.166					0.016*			
Relatedness		137	0.184	0.0145*	–				–				0.082						0.151				0.080					0.142			
Age difference		–274	–0.346	0.0005*	–0.308				0.001*				–						–				–0.232					0.0045*			

* p<0.05. Variables with “-” on the right side of the table were controlled variables in the partial Kr-tests.

† When immediate exchange was excluded, reciprocity controlled for age difference dropped below significance level (*p*<0.1).

**Table 2:** Results of *Kr*-tests: reciprocity of licking and correlations with female–female dyadic variables.

					-Relatedness			-Age difference		-Association index		
Female–female (*N* = 14)	*Kr*		*tau Kr*	*p*	*tau Kr*	*p*	*tau Kr*		*p*	*tau Kr*	*p*	
Reciprocity		201	0.493	0.001*	0.452	0.0005*		0.460	0.0005*	0.446		0.0005*
Relatedness		155	0.290	0.003*	–	–		0.231	0.009*	0.216		0.014*
Age difference		–140	–0.243	0.009*	–0.165	0.047*		–	–	–0.136		0.080

* p<0.005. Variables with “-” on the right side of the table were controlled variables in partial *Kr*-tests.

Hypothesis 2: Social bond. Prediction 2.1 (partially supported): A positive correlation was noted between the frequency of head rubbing and the relatedness of each dyad at the group level. However, the correlation was not significant after we controlled for the effects of age difference and the AI (Table 1). Analysing each actor–receiver sex classs separately, the frequencies of male–male and female-to-male head rubbing were not correlated with relatedness, while the frequencies of female–female and male-to-female head rubbing were correlated with the relatedness of each dyad. In female–female dyads, the correlation between head rubbing frequency and relatedness was not significant after we controlled for either age difference or the AI. The correlation between male-to-female head rubbing frequency and relatedness remained significant after we controlled for the effect of age difference, but it dropped below the level of significance after we controlled for the AI (Table 1). Female licking frequency was significantly correlated with relatedness and the result was not affected when we controlled for age difference and the AI (Table 2).

As in Hypothesis 1, a negative correlation between the frequency of head rubbing and the age difference of each dyad was found at the group level; significant correlations were also found when we analysed each actor–receiver sex class separately, except in male-to-female dyads (Table 1). Female licking frequency was also negatively correlated with relatedness, but the correlation was not significant after we controlled for the AI (Table 2).

**Figure 5:**
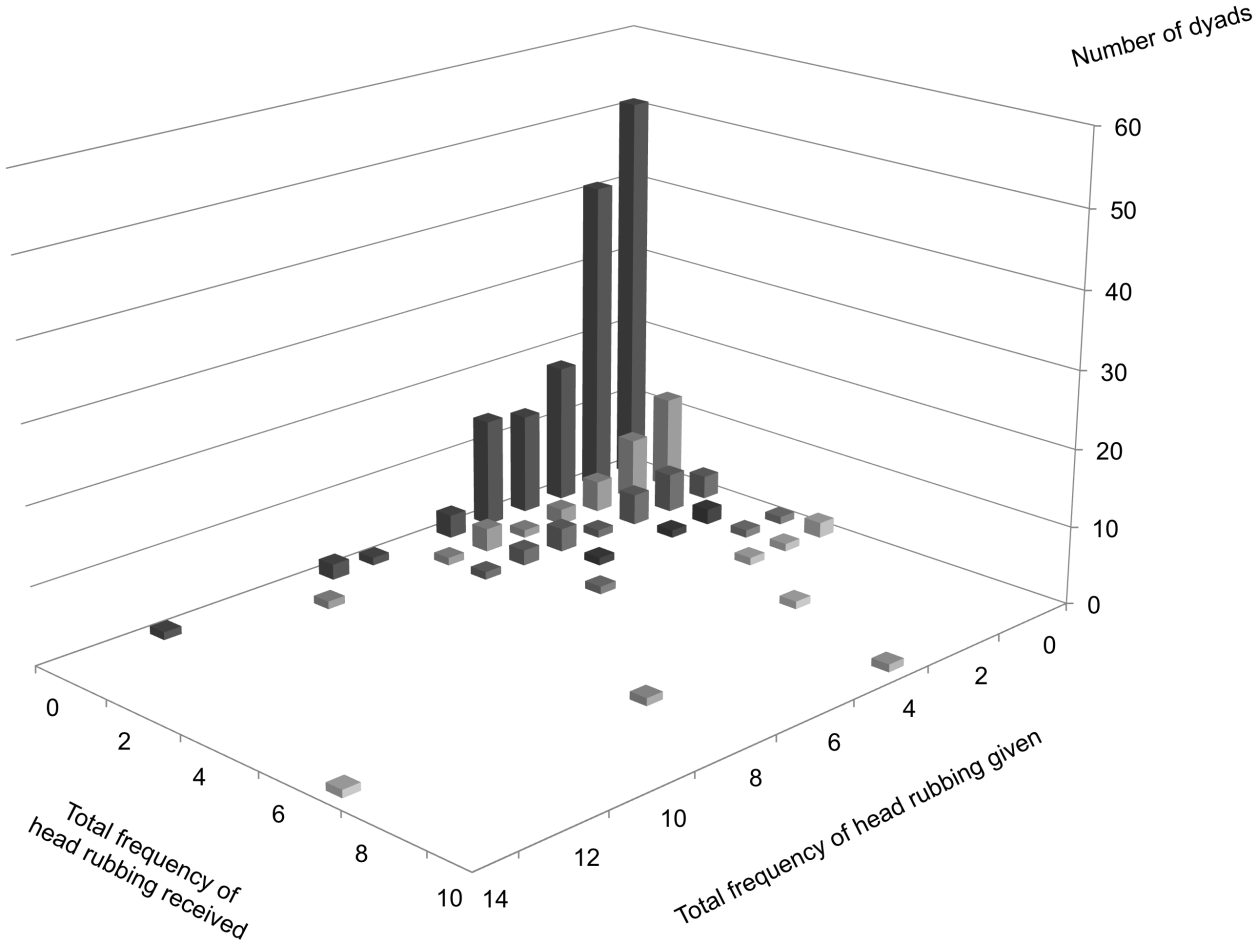
Distribution of head rubbing among all individuals. Each dyad is plotted on a plane according to the summed frequency of head rubbing given by one lion to the other, and vice versa. The cumulative number of dyads is indicated by the height.

**Figure 6:**
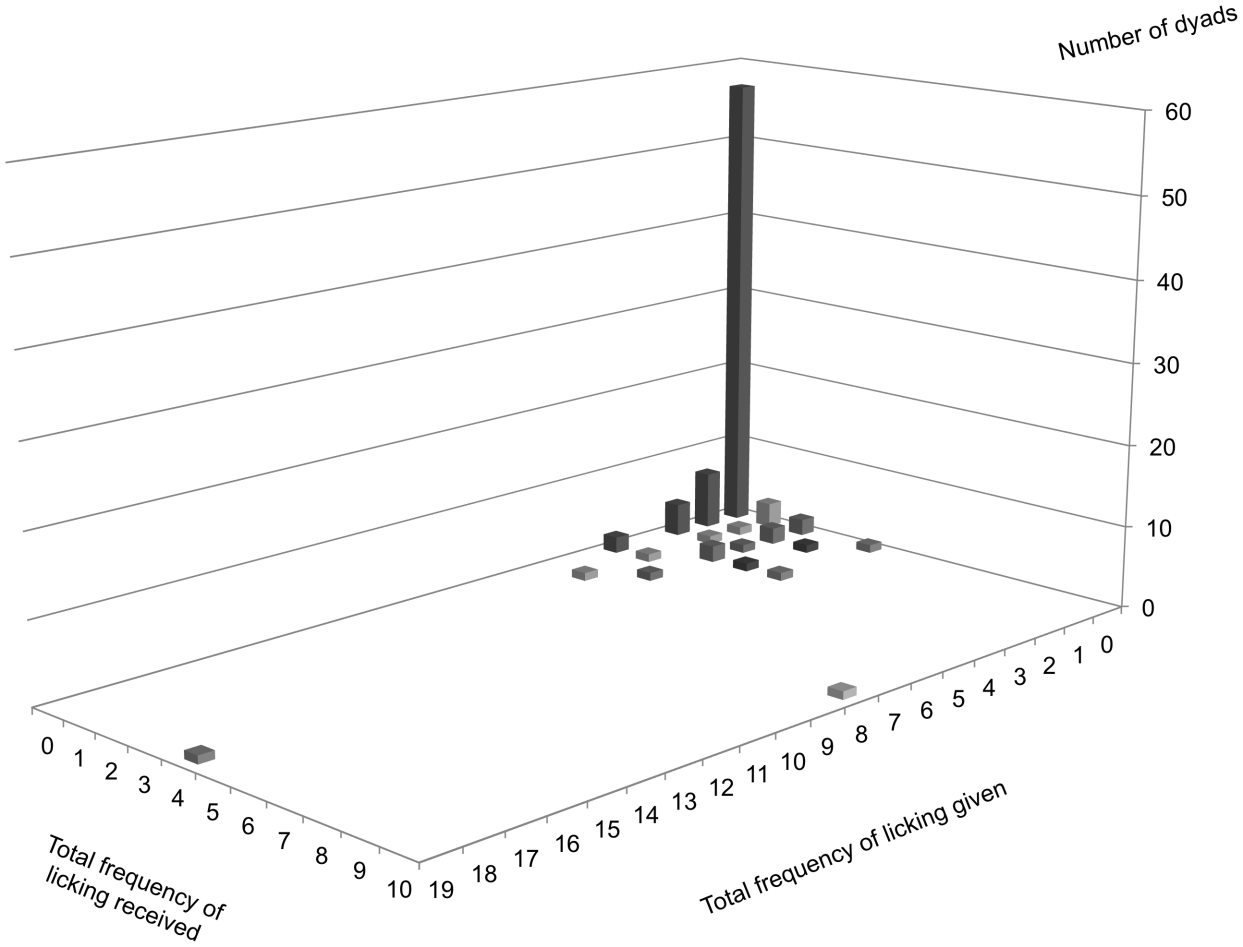
Distribution of licking among females. Each dyad is plotted on a plane according to the summed frequency of licking given by one lion to the other, and vice versa. The cumulative number of dyads is indicated by the height.

Prediction 2.2 (supported): A positive correlation was observed between the frequency of head rubbing given and head rubbing received at the group level (Figure 5). The correlation remained significant after we controlled for the effects of relatedness, age difference and the AI of each dyad. Analysing each actor–receiver sex class separately, while males did not reciprocate head rubbing, interactions in male-and-female and female–female dyads were reciprocal. Again, controlling for relatedness, age difference and the AI did not affect the overall results (Table 1). Likewise, in female–female dyads, the frequencies of licking given and licking received were positively correlated (Figure 6). The correlation remained significant after we controlled for the effects of relatedness age difference, and the AI (Table 2). Excluding the immediate exchange of head rubbing and licking within 10-min time frame did not qualitatively affect the overall results; hence, the results are not presented. The only exception was that overall head rubbing reciprocity dropped below the level of significance when age difference was controlled for (Table 1).

Hypothesis 3: Expression of social status. Prediction 3.1 (a: not supported; b: supported): Affiliative behaviours were frequently observed within same-sex dyads (i.e. head rubbing among males and licking among females) and in female-to-male direction (i.e. head rubbing; see above). Based on this result, these behaviours may function as submission but not as dominance assertions if they contain a signal of relative social status.

Prediction 3.2 (not supported): As in Hypothesis 2, head rubbing was reciprocated at the group level and in male-and-female and female–female dyads (Table 1). Likewise, in female–female dyads, licking interactions were reciprocal (Table 2).

## Discussion

This study showed that the primary function of affiliative behaviours in lions best fits the social bond hypothesis. Head rubbing was reciprocated between members of the group, and its frequency was negatively correlated with dyadic age difference after we controlled for AI. These results indicate that its primary function is to maintain and strengthen social bonds between individuals. Licking was a strongly female-biased behaviour that was reciprocated in female–female dyads. Similar to head rubbing, its frequency was positively correlated with relatedness after we controlled for AI.

We did not find any support for the tension reduction hypothesis. Head rubbing and licking were not more frequent in dyads with a larger age difference whose relationships were predicted to be uncertain. Moreover, we did not observe a significant increase in the frequency of affiliative behaviour at group reunion. These negative results may indicate a reliance on dispersive conflict resolution strategy in this species, as in other social carnivores [4]. However, the latter result should be interpreted cautiously because the separation in this study differs from subgrouping in the wild in many ways, such as having a fixed membership and the availability of acoustic and olfactory signals from separated but near-by individuals. We observed frequent agonistic behaviour immediately after reunion, but this may have resulted not from the stress of reunion itself but from being temporarily restricted in a small area by human intervention; hence, a different coping strategy may have been required.

Support for the social status expression hypothesis was weak and inconsistent. Females directed more head rubbing to males than other females, as the submission hypothesis predicted, but interactions between males and females were reciprocal, although males performed much less head rubbing on the other sex than females. Male–male head rubbing interactions were not reciprocal, but the distribution of agonistic behaviour among them suggested a lack of linear dominance, which is consistent with the pattern in wild populations [24]. Head rubbing and licking in female–female dyads were reciprocal even after controlling for AI and other social variables, which supports the social bond hypothesis as noted above.

The higher frequency of head rubbing in male–male dyads compared to the other sex classses observed in this study indicates strong social bonds in male lion coalitions. In the wild, resident male coalitions engage in high-risk cooperative behaviours, i.e. territorial defence against nomadic male coalitions. Because the numerical odds against intruders is a good predictor of the outcome of a fight, larger coalitions can stay longer in a pride and can enjoy higher per capita reproductive success [46]. The fitness benefits that males gain from the presence of coalition partners is probably the driving force of affiliative relationships in male dyads.

Females showed similar average frequencies for head rubbing and licking toward each other. Since the presence of cubs is known to alter the social behaviours of lionesses, such as territorial defence [56], whether and how cub presence affects relationships between lionesses is an intriguing question that remains to be answered.

Although the male lions in this study exchanged head rubbing more frequently than any of the other sex classs dyads, they rarely licked each other; this is in contrast to females that exchanged both head rubbing and licking. Why was licking rare in males who apparently formed strong social bonds, as judged by head rubbing frequency? One possibility is that licking is more easily triggered in females because it is originally adopted from the behavioural repertoire of maternal care. This may also be the case in female contact swimming behaviours in bottlenose dolphins. Connor et al. [57] suggested that contact swimming was originally a maternal behaviour with calves to assist their locomotion and has subsequently been employed to signal social bonds in females. However, male cheetahs within a coalition groom each other but do not engage in head rubbing [20], which runs counter to this hypothesis. Due to the small number of males in a pride, male–male social interactions have been poorly described in previous studies [24], [32]. Clearly, more observation data, especially from wild populations, are essential to account for sex differences in affiliative behaviours.

The pattern of affiliative behaviours demonstrated in this study was strongly affected by the sexes of the participants and relationship quality. Similarly, the pattern of greeting in spotted hyenas is strongly affected by the sexes of the participants (i.e. they are more frequent in females, which is the dominant sex in that species) and the relationship quality of interacting dyads (i.e. more frequent in close associates and coalition partners) [4]. Greeting in spotted hyenas has a cost in terms of exposing vulnerable genital areas to interactions with partners. In the case of head rubbing in lions, it seems to involve less conspicuous costs, so the behaviour itself may be less effective as an honest signal and olfactory information may play a complementary role [34], as in other social carnivores (e.g., [58], [59]). Group-specific odour in social carnivores may result from a shared bacterial community of the members, mediated by coexistence in the same space, frequent bodily contact and/or consistent scent marking of the same sites [59], [60]. Social behaviour of lions fit all of these conditions [24]. The olfactory aspects of the bodily contact of lions need further investigation including the recording of scent marking behaviour and/or chemical analysis.

Since this study was conducted on a group of lions in captivity, the extent to which the results reflect affiliative behaviour patterns in the wild is unsettled and the interpretation requires caution. It was preferable to investigate greeting patterns in a larger number of individuals, which should affect the power of the statistical tests. The sex ratio and size (6 adult males and 12 adult females) of this group fall within previously reported ranges for wild prides. One critical difference is probably the lack of dispersal that created a group composition in which adult males are related to adult females, which differs from natural conditions. With its wide distribution, the lion shows considerable variation in group size, group composition, and social behaviours in relation to habitat type and prey distributions [22], [23]. In savannah woodlands in Kruger National Park, for example, males disperse at older ages (40 months on average, maximum 60 months) compared to individuals living in plains-like habitats, and the absence of resident pride males lasts on average 12 months and up to 15 months [61]. Therefore, at least in some wild populations, the temporary residence of an adult male coalition in the natal pride, similar to the condition in the studied group, is possible. However, we acknowledge that our observations were strongly affected by the captive conditions in several ways. Social mammals in captivity allocate more of their time to social interactions and less to foraging compared to wild populations. Decreased inter-individual distance and the lack of food competition have kept the studied group of lions from replicating the fission–fusion dynamics observed in wild populations. Although the animals in this study did not show stereotypies, carnivore species with larger home ranges, including lions, are more likely to exhibit stereotypies in captivity than species with smaller home ranges [62]. To determine whether captivity and other group-specific variables affect the dynamics of social interactions among individuals, additional studies that focus on the details of social interactions in both captive and wild lion groups are necessary.

This study showed that intragroup affiliative behaviours in lions help maintain social bonds with preferred partners based on kinship and age proximity. In wild populations, the distribution of affiliative social interactions may reflect partner choice in subgroups, as in spotted hyenas [4], another fission–fusion group-living carnivore. In a recent analysis of extensive field data, Mosser and Packer [25] showed that the pattern of subgrouping in wild lions is affected by the interaction between group size and the risk of intergroup territorial conflict. Similarly, the pattern of affiliative interactions may reflect changes in the costs and benefits of sociality caused by immediate behavioural contexts. To test this idea, future studies should focus on affiliative behaviours in more specific contexts. For example, are strongly bonded dyads better coordinated than weakly bonded ones in synchronous behaviours [42], such as chorus roaring and responses to approaching intruders in territorial defence? Are affiliative behaviours distributed more evenly when bonds should be extended to the whole group, such as before hunting a large and dangerous prey [63] or after intergroup territorial conflict [64]? Such future research would greatly enhance our understanding of intragroup relationships in this highly social species.
